# Single-cell computational machine learning approaches to immune-mediated inflammatory disease: New tools uncover novel fibroblast and macrophage interactions driving pathogenesis

**DOI:** 10.3389/fimmu.2022.1076700

**Published:** 2023-01-04

**Authors:** Douglas Fritz, Jun Inamo, Fan Zhang

**Affiliations:** ^1^ Medical Scientist Training Program, University of Colorado School of Medicine, Aurora, CO, United States; ^2^ Division of Rheumatology, Department of Medicine, University of Colorado School of Medicine, Aurora, CO, United States; ^3^ Center for Health Artificial Intelligence, Department of Biomedical Informatics, University of Colorado School of Medicine, Aurora, CO, United States

**Keywords:** computational biology, machine learning, single-cell omics, spatial transcriptomics, immune-mediated inflammatory disease, rheumatoid arthritis, fibroblast-macrophage interaction

## Abstract

Recent advances in single-cell sequencing technologies call for greater computational scalability and sensitivity to analytically decompose diseased tissues and expose meaningful biological relevance in individual cells with high resolution. And while fibroblasts, one of the most abundant cell types in tissues, were long thought to display relative homogeneity, recent analytical and technical advances in single-cell sequencing have exposed wide variation and sub-phenotypes of fibroblasts of potential and apparent clinical significance to inflammatory diseases. Alongside anticipated improvements in single cell spatial sequencing resolution, new computational biology techniques have formed the technical backbone when exploring fibroblast heterogeneity. More robust models are required, however. This review will summarize the key advancements in computational techniques that are being deployed to categorize fibroblast heterogeneity and their interaction with the myeloid compartments in specific biological and clinical contexts. First, typical machine-learning-aided methods such as dimensionality reduction, clustering, and trajectory inference, have exposed the role of fibroblast subpopulations in inflammatory disease pathologies. Second, these techniques, coupled with single-cell predicted computational methods have raised novel interactomes between fibroblasts and macrophages of potential clinical significance to many immune-mediated inflammatory diseases such as rheumatoid arthritis, ulcerative colitis, lupus, systemic sclerosis, and others. Third, recently developed scalable integrative methods have the potential to map cross-cell-type spatial interactions at the single-cell level while cross-tissue analysis with these models reveals shared biological mechanisms between disease contexts. Finally, these advanced computational omics approaches have the potential to be leveraged toward therapeutic strategies that target fibroblast-macrophage interactions in a wide variety of inflammatory diseases.

## Introduction

Immune-mediated inflammatory diseases (IMIDs) are roughly categorized by abnormal or maladaptive inflammation of specific tissues within the human body and are thought to affect nearly 3% of the population (1). The increasing prevalence of IMID diseases such as inflammatory joint disease and inflammatory bowel disease and their respective subphenotypes has driven additional research into the genetic and immunogenomic mechanisms involved in their development, progression, and treatment (2, 3). Because of fibroblasts’ ubiquity in the lining of interior surfaces of the human body and their role in mediating the extracellular matrix, fibroblasts have recently become an area of intense research and a key component of the study of IMIDs (4, 5).

Fibroblasts play a critical role in inflammatory disease by directing or suppressing the inflammatory cascade and repair at sites of injury or invasion through the release of cytokines and other effector molecules (6). In addition, bone and extracellular metabolic pathways are also involved in pathogenesis: activated fibroblasts produce receptor activator of NF-κB ligand (RANKL), which promotes differentiation of osteoclast precursors into bone-resorbing osteoclasts, leading to bone erosion in Rheumatoid Arthritis (RA) (7). They also produce metalloproteinases such as MMP-1 and MMP-3, which cause cartilage degradation. As a result, understanding these and other intercellular communications between fibroblasts and surrounding cell types is an area of rapid research and critical to understanding the microbiological contexts of IMID toward developing new drug targets (4, 8, 9). While the communications between fibroblasts and immune cells such as macrophages is a common focus of cancer research (10), applying this framework to the study of IMIDs has also proved consequential in determining an approach to treatment (11). However, these interactions are often highly tissue-specific and microenvironment-specific and require precise study using high-resolution single-cell multiomic technologies.

The challenge of mapping these cellular interactions in inflammatory microenvironments, then, becomes one that is highly dependent on advances in single-cell transcriptomics, single-cell multimodal techniques, and recent single-cell spatial transcriptomics (12, 13). While collecting omics data at the single-cell level has been commonly applied to discrete cellular suspensions *via* microfluidics (12, 14–17), collecting single-cell spatiotemporal data and prevailing tissue microenvironment intact has proved elusive and high-throughput techniques with these capabilities are hotly anticipated by the field (18). At present, technical resolution remains a challenge to mapping the complex tissue intercellular interactions thought to be pivotal toward IMID treatments, but this challenge is exacerbated by the enormous volumes of data that near-single cell omics technologies create. This review outlines how computational methods including machine learning and deep learning approaches are used to analyze high-dimensional data from existing single-cell technologies, which expand the capabilities and resolution of these experimental approaches to uncover novel pathways in fibroblasts. Then, this paper summarizes the computational approaches to cell-cell interactions that can be used to uncover these interactomes underlying IMIDs using single-cell transcriptomics and spatial transcriptomics, respectively. Further, opportunities and challenges of integrating single-cell profiles from multiple tissue sources to reveal shared and unique pathogenic pathways are described. Lastly, we explore the potential for developing therapeutic approaches that target pathogenic fibroblast and macrophage interactions.

## Fibroblasts play important roles in different inflammatory disease tissue pathology

Long thought to be relatively homogeneous in nature, recent discoveries (18–22) have uncovered a likely vast number of fibroblast subpopulations with discrete markers that have been implicated in mediating inflammation and damage in different IMIDs (23–25). In RA synovial tissue, Zhang et al. analyzed the synovial tissues from patients with RA and osteoarthritis (OA) using a multi-technology-approach by integrating single-cell RNA-seq (scRNA-seq), mass cytometry, and bulk RNA-seq data to identify robust and biologically meaningful cell-state clusters (26). An integrative computational strategy was developed based on canonical correlation analysis (CCA) to align datasets from different technologies into a joint low-dimensional space by maximizing the correlation between them, which elucidated significant sublining fibroblast phenotypes, CD34+ (SC-F1), HLA-DRhi (SC-F2), and DKK3+ (SC-F3), and a type of CD55+ lining fibroblasts (SC-F4). In parallel, Croft et al. used single-cell transcriptomic analysis in a mouse model to untangle two pathologically distinct RA fibroblast subsets FAPα+THY1+ and FAPα+THY1-. Deletion of fibroblast-activation-protein-alpha-positive (FAPα^+^) fibroblasts suppressed both inflammation and bone erosions in mouse models (27). Separate studies have revealed pathological functions of stromal cells in other IMID tissues, including the gut of Ulcerative Colitis (UC) (8), the ileum of Crohn’s Disease (CD) (28), and the lungs of systemic sclerosis (29) patients, respectively. Interestingly, similar THY1+ fibroblasts are revealed in inflamed CD ileum, and an activated fibroblast phenotype with a strong cytokine-chemokine expression profile in this tissue may contribute to the resistance to anti-TNF therapy. In parallel, inflammatory fibroblasts that highly expressed IL11 and IL24, were identified at 189-fold levels in inflamed gut compared to non-inflamed/healthy gut; this phenotype also expressed cancer-associated fibroblast markers, including FAP and WNT2, indicating the important pathology underlying multiple disease contexts (8).

**Figure 1:**
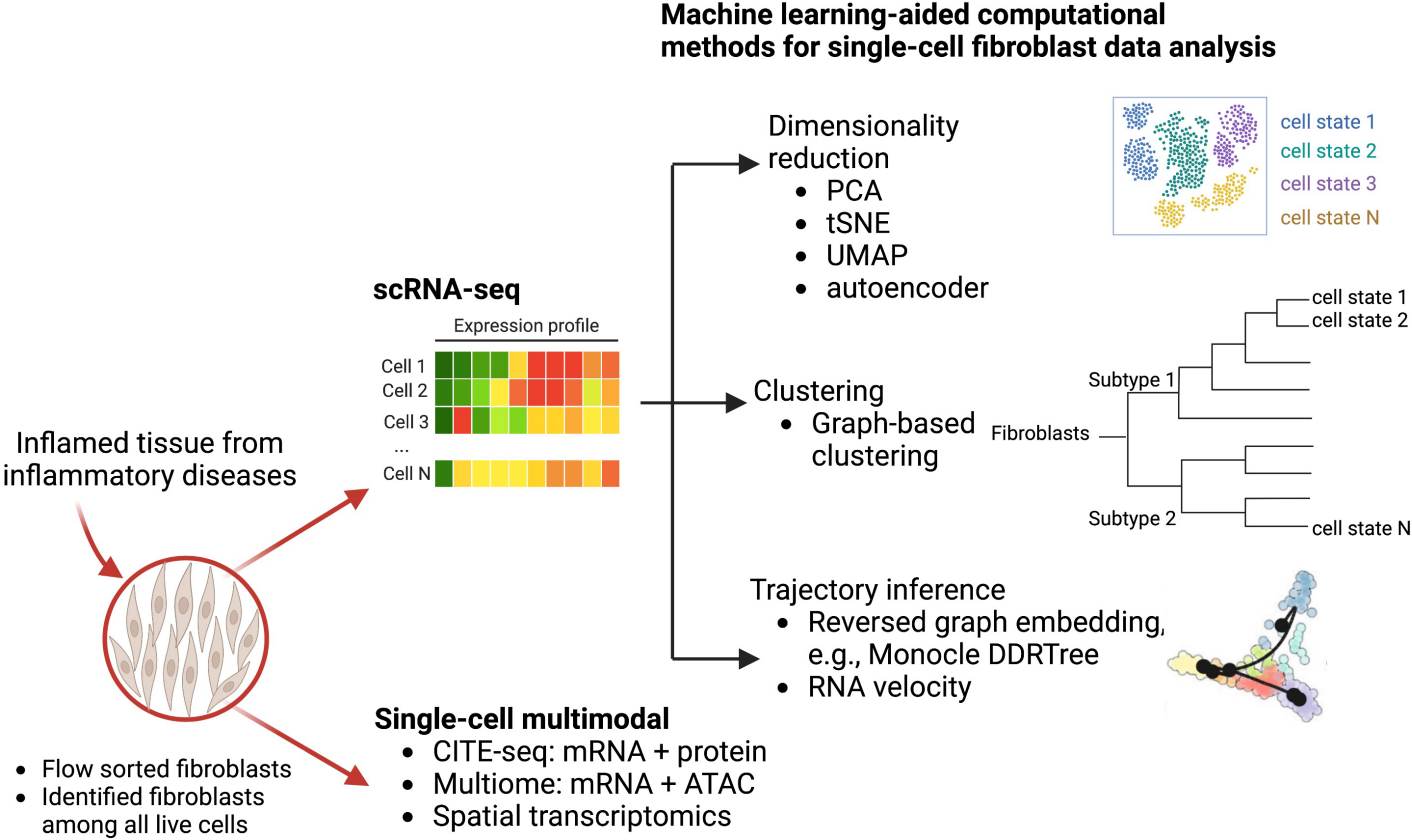
Computational machine learning algorithms that drive single-cell transcriptomics and other multimodal data analysis to study fibroblast heterogeneity. Disaggregated cells are sequenced by either multimodal technologies or scRNA-seq technologies to generate a large dataset of thousands of variables that can be modeled and analyzed *via* machine learning approaches. Graphical exploration of gene expression variation across cells can be done through clustering for dimensionality reduction. Continuous gene expression changes for specific cell differentiation processes can be modeled with trajectory inference.

In these studies, several computational methods are used to facilitate the single-cell transcriptomic analysis to reveal fibroblast heterogeneity (**Figure 1**). In particular, dimensionality reduction techniques including principal component analysis (PCA) and non-linear tSNE are standard approaches to identify meaningful biological variation. Additionally, graph-based clustering techniques group fibroblasts with similar transcriptomic profiles together. To better account for non-linear geometry and time components in the single-cell data, trajectory inferences have been widely used to allocate and order cells into lineages as pseudotime gradients. Pseudotime reflects continuous changes in expression to quantitatively capture a biological progression, such as cell differentiation. Based on global topology theory, several computational methods have been developed, including Monocle, which is built based on DDRTree (Discriminative dimensionality reduction *via* learning a tree) (30, 31). To predict the future state of individual cells, RNA velocity algorithms (32) estimate the time derivative of the gene expression state by distinguishing unspliced and spliced mRNAs from single-cell transcriptomic data. These trajectory analyses have been deployed to analyze fibroblast lineages to reveal a NOTCH3 signaling gradient in RA synovial fibroblasts (33).

As the recent development of single-cell multimodal technologies, single-cell joint modelings are used to provide further insights into mesenchymal cell heterogeneity using single-cell multimodal data, including CITE-seq that quantifies gene and protein surface expressions simultaneously (34), single-cell multiome that profiles gene expression and open chromatin from the same cells, and spatial transcriptomics (ST) that provides spatial information to the gene expression. Multimodal data integrations provide additional biological perspectives, through the combination of proteomics (CITE-seq), epigenetics (multiome), or spatial locations (ST), in addition to transcriptomics, which can reveal novel immunological or disease-driven insights. A very recent study with a collaborative effort from AMP (Accelerating Medicines Partnership) RA/SLE network used CITE-seq to reveal 10 distinct stromal populations and emphasized which of these populations are expanded in a particular patient group (35). Using three-dimensional spatial transcriptomics, Vickovic et al. uncovers colocalization of THY1+ fibroblast and synovial macrophages in seropositive RA synovium samples (36).

## Single-cell transcriptomics-driven computational methods reveal predicted interactomes between fibroblasts and myeloid cells

**Figure 2:**
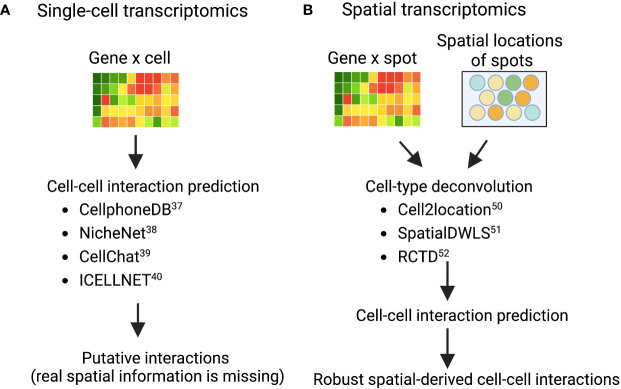
Validated computational packages to predict cell-cell interactions using **(A)** single-cell transcriptomics and **(B)** spatial transcriptomics, respectively Cell-cell interaction prediction algorithms **(A)** are quite adept at mapping interactions from single-cell transcriptomics where tissue architecture information was lost in sample preparation. As a result, interactions revealed in analysis are theoretical and merely suggest that cells co-located in the sample tissue. However, combining these interaction-prediction-algorithms with spatial cell-type deconvolution modeling **(B)**. The sample tissue’s spatial architecture is conserved by modeling cells that are both co-locating in the tissue and interacting.

Deciphering cell–cell communications from gene expression is an area of intensive research (37). Many computational methods have been developed based on ligand-receptor expression patterns between cell types, such as fibroblasts and myeloid cells (macrophages, monocytes, neutrophils, and dendritic cells). Examples of these techniques are: CellphoneDB (38), NicheNet (39), CellChat (40), and ICELLNET (41), each of which took a slightly different methodology to predict potential cell-cell interactions in scRNA-seq data (**Figure 2A**). CellphoneDB was first demonstrated (38), and has since been updated through multiple iterations of a ligand-receptor mapping tool (42). NicheNet incorporates prior knowledge on gene regulatory pathways to generate a biologically meaningful pathway that propagates the signal from a ligand, through receptors, signaling proteins, and transcriptional regulators to the targeted genes from cell types of interest. CellChat, on the other hand, uses network analysis, and identifies complex patterns in the data from skin or other tissues; while ICELLNET calculates a communication score to predict interactions and reveals hypothesized interactions that can be verified experimentally. In short, these cell-cell interaction prediction methods are widely used to prioritize putative interactions between fibroblasts and other immune cells, such as macrophages, from different disease contexts, including tumor (10), fibrosis (43), and cardiovascular disease (44). As fibroblasts and macrophages play indispensable roles in the tissue destruction of IMIDs, disentangling the fibroblast-myeloid interactions in each IMID disease context is still forthcoming (45). A recent single-cell driven research approach identified a MerTK+ macrophage phenotype in synovial tissues and revealed that a low frequency of this phenotype in RA remission was associated with increased risk of disease flare (46). Further examination of which pathogenic fibroblast phenotypes could interact with the MerTK+ or other inflammatory and anti-inflammatory macrophage phenotypes is needed. In another IMID, a single-cell transcriptomics and histopathology approach to inflammatory bowel disease (IBD) revealed an IL-1+ driven fibroblast-neutrophil interaction in a subset of patients with IBD that did not respond to therapies (47), which highlights another fibroblast-neutrophil IL-1 signaling pathway for ulcerating disease.

## Novel computational approaches to spatial transcriptomics reveal spatial interactions across cell types

While CellPhoneDB and similar packages are useful for revealing interactomes in scRNA-seq data, decomposing the data spatially within tissues remains a challenge. Spatial Transcriptomics (ST) technology development and the improvement of its resolution enabled the identification of cross-cell type interactions from both gene expression co-varying patterns and spatial information. The widely used commercialized ST technologies include 10X Visium, Nanostring GeoMX, and single-molecule fluorescent *in situ* hybridization (smFISH)-based technology such as MERFISH commercialized by Vizgen (48, 49). These ST datasources require new computational algorithms to infer biologically meaningful findings and to spur further widespread adoption of these techniques across IMIDs.

Given the constraint of greater-than-single-cell resolution of many commercialized ST technologies, more than 16 computational methods have been developed to perform cell-type deconvolution for ST data to infer single-cell interactions (50). As a result (**Figure 2B**), Cell2location (51), SpatialDWLS (52), and RCTD (53) are particularly powerful approaches that perform cell-type deconvolution. Cell2location is developed to integrate scRNA-seq data from an adjacent tissue slice with the spatial information from the microarray, which can effectively identify the spatial co-occurrence of diverse cell types in complex tissues such as lymph nodes (51). SpatialDWLS adapts the idea of dampened weighted least squared to infer cell-type composition while minimizing the overall relative error rate. RCTD fits the raw counts using Poisson-based statistical model to leverage cell-type mixtures while accounting for artifact from sequencing platforms. Many groups have demonstrated that these techniques work well in tumors from the well-characterized organs such as brain (54, 55), but in some heterogeneous or not well-characterized tissue structures such as synovium and kidney tissues it remains to be evaluated whether these techniques can be deployed. Ongoing efforts from the AMP-AIM (Accelerating Medicines Partnership-Autoimmune and Immune-Mediated Diseases) network are actively testing multiple ST technologies on IMIDs disease tissues. We look forward to both the deployment of newer and higher-resolution techniques that might be better suited to these IMID tissue-structures and to further benchmarking of existing and forthcoming computational models. With these efforts, more in-depth spatial-aware interactions between fibroblasts and myeloid cells will be revealed using ST data with the assistance of more robust computational methods.

## Cross-tissue single-cell integrative analysis reveals shared mechanisms

**Figure 3:**
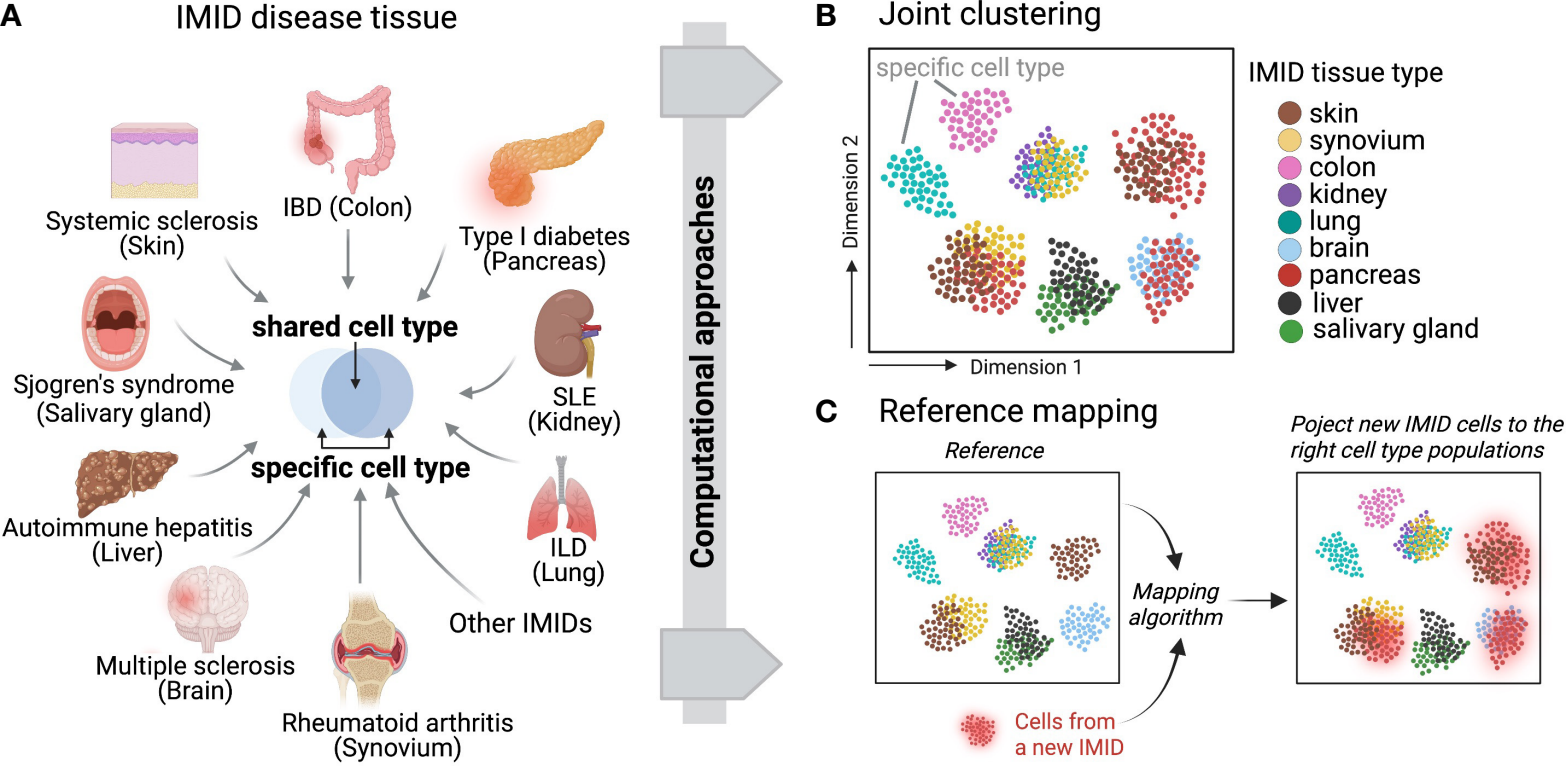
Across IMID single-cell integrative analysis. **(A)** Multiple IMIDs (organ systems) where inflammatory and pathogenic cells display both heterogeneity and similarity between tissue types and within disease contexts. Computational approaches can be deployed to disentangle the shared and specific pathways between IMIDs while also controlling for tissue heterogeneity. Two main computational frameworks include **(B)** integrative and joint clustering analysis and **(C)** reference mapping approach. **(B, C)** indicate the low-dimensional projections of cells across IMIDs.

Recent developments of computational integration algorithms enable the cross-tissue, cross-disease comparisons for IMIDs to reveal shared mechanisms and pathways using single-cell datasets (**Figure 3A**). To facilitate unbiased integrative analysis, two major types of methods have been developed including joint clustering and reference mapping (**Figures 3B, C**). In joint clustering, batch correction methods, such as soft clustering-based mixed effect models (56), canonical correlation analysis (57), mutual nearest-neighbors and manifold learning (58), have been developed to enforce projecting the cells from different tissues, donors, and clinical cohorts into a joint low-dimensional embeddings (i.e. multiple variables captured on a 2D graph) (**Figure 3B**). Additionally, recent single-cell reference mapping methods, including PCA-based approaches, transfer learning, and autoencoder, enable an automatic way to map query cells to an existing reference with cell-type annotations (59–62) (**Figure 3C**). These offer a more efficient framework to compare query cell phenotypes with an existing cell reference. A recent study performed joint clustering analysis to reveal two shared pathogenic phenotypes of fibroblasts, a CXCL10+ CCL19+ inflammatory fibroblast phenotype localizing to a T cell enriched niche and a SPARC+ COL3A1+ fibroblast phenotype localizing to a perivascular niche, from four chronic inflammatory diseased tissues including lung, intestine, salivary gland, and synovium (63). Another study built fibroblast atlases using around 230,000 fibroblasts across 17 mouse tissues and revealed that many fibroblast transcriptional states were conserved between humans and mice (64). In parallel, we identified shared inflammatory macrophage phenotypes from five inflamed tissues, including synovium, ileum, colon, lung, and kidney (65). These recent cross-tissue single-cell computation-driven transformative research open new possibilities beyond well-known cell types and pathways.

However, key aspects of these techniques need to be validated to interpret single-cell integrative results more precisely regarding disease-specific implications. First, sufficient power is required to reveal statistical significance of associating single-cell results with clinical metrics and demographic features. A large-scale cohort with balanced disease and healthy controls and well characterized medications is ideal. Second, reproducible analysis of computational pipelines is sometimes neglected. For example, it remains largely under-explored whether the same common fibroblast phenotypes can be recapitulated in another clinical cohort. It is possible that the tissue-specific fibroblast phenotypes found in a certain diseased context are actually due to unbalanced cell numbers in the cross-sample analysis. Yet, as an active computational and systems immunology area, we expect these computational machine learning algorithms and future developments will boost the transformative research to elucidate shared pathogenic pathways and treatment areas.

## Opportunities for developing therapeutical strategies targeting fibroblast and macrophage interactions for inflammatory diseases

Remarkable recent advances in understanding the molecular pathogenesis of IMIDs have elucidated relevant pathophysiological pathways and therapeutic targets. Inhibition of TNF and IL6 signaling, for example, has shown some efficacy in treating various IMID contexts, including RA and ulcerative colitis (66–68). Similarly, in systemic sclerosis, in which fibroblasts and macrophages are deeply involved in the pathogenesis of lung damage, IL6 blockade delayed decline in key lung function measures compared with the placebo groups in a double-blind phase II randomized clinical trial (69, 70). Additionally, although strong evidence from experimental models and human data *in vivo* and *in situ* suggested potential of anti-IL17 blockade as a therapeutic target in RA (71, 72), psoriasis (73), and spondyloarthritis (74), strong efficacy for RA and other similar diseases has not shown in comparison to placebo (75). Thus, ineligible patients with IMIDs still suffer from progressive functional disability from a substantial burden of lifelong treatment—highlighting the existence of the remaining pathological molecular signatures and the urgent need to link them to targeted core-pathogenic phenotypes, such as mesenchymal and immune cell interactions at the site of inflammation.

**Figure 4:**
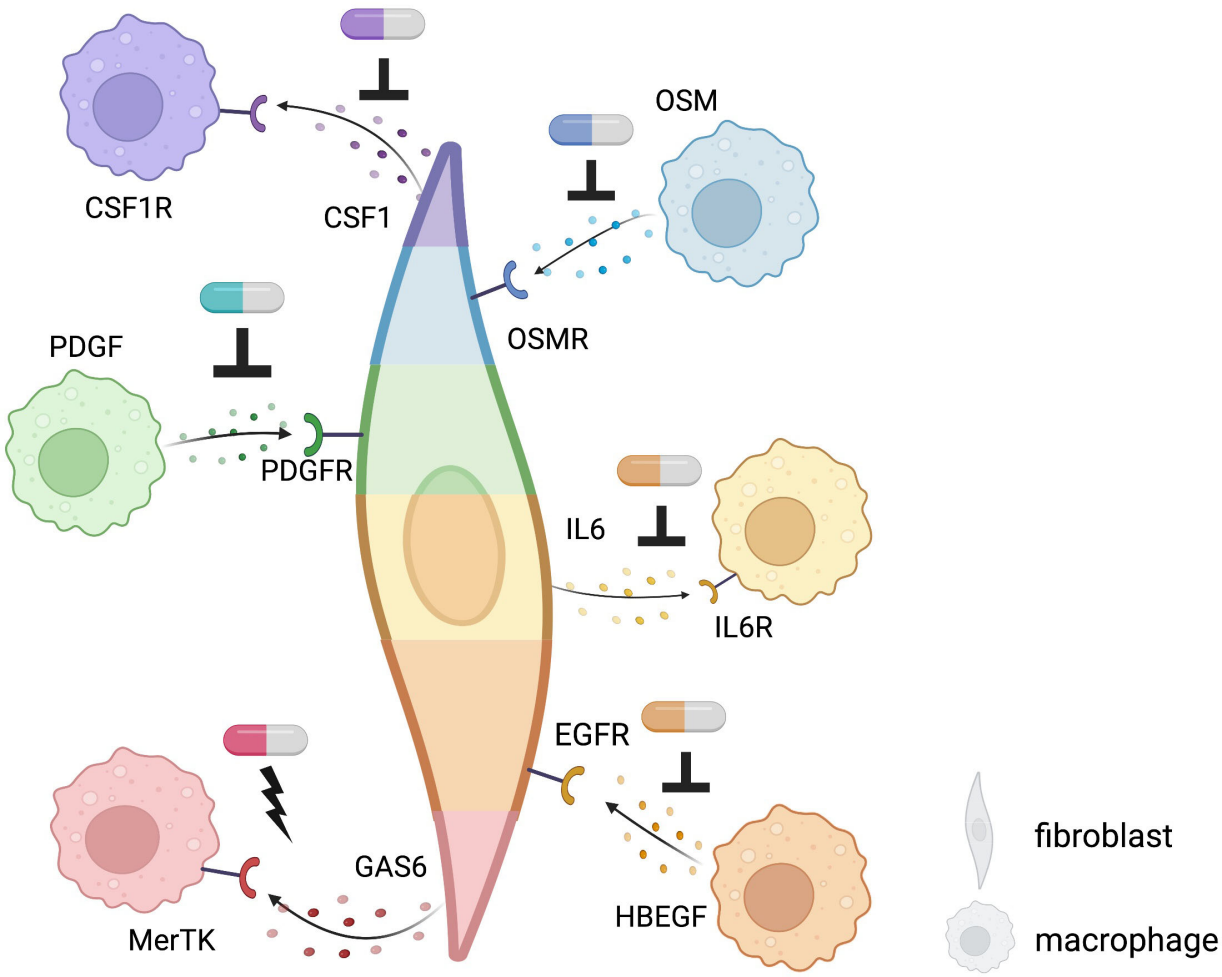
Potential targets of fibroblast-macrophage interacting revealed by single-cell computational methods using existing receptor-ligand pairs in IMIDs. Targets of novel therapeutics: potential sites of inhibition are indicated by an upside-down T while sites of activation are indicated by a lightning bolt. Different colors of each fibroblast and macrophage indicate different phenotypes of each cell type. Pill icons indicate potential, experimental, or existing therapeutics.

A precise understanding of fibroblasts and macrophages, major tissue components in IMIDs, may promote the development of novel therapeutic targets. Key interactions based on the well-known pathways and new mechanisms revealed by single-cell computational omics are summarized in **Figure 4**. Fibroblasts and macrophages produce CSF1 (Colony Stimulating Factor 1) and PDGFs (platelet-derived growth factors), respectively, and bind to each other’s receptors to promote survival, maintenance, and proliferation, forming a synergistic loop in a steady-state (76, 77) and upon activation (78, 79). In a radiation-induced pulmonary fibrosis model, depletion of tissue-infiltrating macrophages, but not alveolar macrophages, using a clinically available CSF1R neutralizing antibody ameliorated fibrosis (79). Similarly, Aran et al. demonstrated that inhibition of Pdgf-aa produced by the inflammatory macrophage identified by single-cell sequence suppressed fibroblast growth in bleomycin-induced lung fibrosis in mice (78). Accumulating evidence of clinical efficacy of inhibition of tyrosine-kinase, which is a downstream molecule of CSF1R and PDGFR, for IMIDs suggests that targeting the interactions between fibroblasts and macrophages are highly promising strategies towards individualized and targeted treatments of IMIDs (80–82). Using single-cell transcriptomics, Kuo et al. reported that a particular HBEGF (Heparin Binding EGF Like Growth Factor)+ inflammatory macrophage phenotype was induced by fibroblasts and TNF in RA synovium, subsequently promoted fibroblast invasiveness (83). They also found that this interaction was inhibited by anti-EGFR (Epidermal Growth Factor Receptor) antibody, which decreased pathogenic fibroblast invasiveness in the destruction of cartilage and bone. Further experimental evidence and case reports support the potential of EGFR as a promising therapeutic target for RA (84–86).

In IBD, anti-TNF agents bring about clinical response in about two-thirds of patients, but around 30% of patients are resistant to treatment (87, 88). Using single-cell omics, inflammatory fibroblasts and inflammatory monocytes were identified to be expanded in inflamed colon lesion and expressed Oncostatin M (OSM) and OSM receptor (8), respectively, which is associated with anti-TNF response, suggesting that the inflammatory fibroblasts and monocytes might be implicated in OSM-mediated anti-TNF resistance (89). Methods that not only inhibit interactions but also exploit interactions with anti-inflammatory effects may be promising therapeutic targets. The addition of GAS6 from THY1+CXCL14+ sublining synovial fibroblasts, reduced proinflammatory cytokines produced by MerTK+ macrophages in synovial tissues of RA (46). On the flip side, GAS6 and MerTK are reported to be overexpressed in tumor which could promote tumorigenesis (90–92). It is necessary to clarify the difference between malignancy and inflammation in this pathway and to examine what route of administration, such as intra-articular injection, is appropriate for therapeutic targeting.

So far, no drugs targeting specifically fibroblasts have been approved by the U.S. Food and Drug Administration (FDA). Thus, identification of promising across cell-type interactome targets, such as fibroblasts and macrophages in inflammatory lesions, using single-cell technologies combined with powerful computational tools could lead to the development of effective therapeutics for IMIDs, as in the area of oncology (43). If markers characteristic of disease-specific cell types that play a central role in the pathogenesis utilizing single-cell high granular results can be identified, more accurate therapeutic agents can be developed to minimize the adverse event and improve precision medicine.

## Future directions

Most of these computational methods described above can be generalized to many inflammatory disease studies. For example, t-SNE and UMAP are used widely for dimensionality reduction analysis for many IMID research projects. Additionally, techniques like graph-based clustering and trajectory analysis are umbrella classifications that are highly modified and adapted depending on data type and context. Yet, each computational method may have specific limitations derived from disease tissue (e.g., tissue disaggregation approaches) or technology (e.g., high dropout rates, non-single-cell resolution in the recent spatial transcriptomics) when applied across multiple IMID contexts. This review summarizes the most recent computational advancements and major novel disease-specific findings combined with cutting-edge single-cell techniques to IMIDs, so we expect more generalized applications of these interdisciplinary approaches along with computational machine learning algorithms can be adapted to more understudied IMIDs. Taking advantage of the power of these computational algorithms helps generate novel cell phenotype and highlight theoretical cell-cell interactions in humans. More in-depth functionally validations (e.g., knockout specific target, *in vivo* or *in vitro* stimulation) are needed, however, to determine the function mechanisms of these interactions and disease etiology in human and non-human models.

To develop personalized treatment for IMIDs, it is necessary to identify the cell-type that forms the core of the pathogenesis in stratified patient groups. For example, analysis of bulk RNA-seq from skin lesions from systemic sclerosis patients using cell-type deconvolution methods demonstrated that certain types of serum autoantibodies were associated with dysregulated molecular pathways as well a predictable abundance of fibroblasts and macrophages at the skin lesion (93). In RA, bulk RNA-seq studies defined three histological subgroups or “pathotypes”: lympho-myeloid, diffuse-myeloid, and pauci-immune (94, 95). The myeloid signature is associated with response to TNF inhibition, while the pauci-immune group, predominated with fibroblasts, is associated with refractory to multi-drugs (94). This indicates that mesenchymal cell compartment is a key population for further study using higher-resolution technologies, such as single-cell omics, as it is unclear whether the specific high-granularity pathogenic subphenotypes underlying these pathotypes are targetable therapeutically.

More recently, Zhang et al. demonstrated that in-depth stratification of RA synovial biopsies based on single-cell multimodal integrative analysis combined with covarying neighborhood analysis can associate cellular heterogeneity to stratified RA synovial phenotypes. Specifically, RA synovial heterogeneity was classified into six distinct subgroups or “cell type abundance phenotypes” (CTAPs) based on major cell-type abundance (35): 1) endothelial, fibroblast, and myeloid cells, 2) fibroblasts, 3) T cells and fibroblasts, 4) T and B cells, 5) T and myeloid cells, and 6) myeloid cells. Three of the CTAPs have associations with fibroblast and their immune interaction abundances suggesting that different patients, even with the same disease, have different tissue phenotypes at the core of their pathology, and accordingly, different molecules to be targeted for therapy. Notably, CTAPs are associated with disease-relevant cytokines, histology, and serology metrics, which indicates that the CTAP classification schema could guide appropriate targeted therapeutic treatment.

Yet, knowledge in this area is limited by the availability of biopsies from inflamed lesions derived from IMID patients. To address this, better single-cell power analysis of study design, demographic information, and technical confounders need to be considered to strengthen biologically relevant findings. Moreover, identifying the right computational and machine learning approaches is critical for downstream analysis. For example, more reproducible single-cell analytical methods with open-source code and well-benchmarked machine learning methods regarding stability and accuracy need to be provided and further improved. Given the complexity of the immunological questions, new computational and disease-driven tools using AI approaches may provide further insights into disease etiology. In all, comprehensive characterization of cellular and molecular heterogeneity in inflamed lesions using single-cell computational machine learning approaches will enhance our understanding of disease heterogeneity, which will provide a promising way to stratify patient cohorts to optimize personalized therapies for IMIDs.

## Author contributions

DF and FZ conceived of the idea for this review, compiled literature, and reviewed relevant manuscripts. DF led the writing of the manuscript as was supported by JI and FZ on particular subsections. JI and FZ lead figure design. All authors provided critical feedback and helped shape the research and manuscript. All authors contributed to the article and approved the submitted version.
